# The impact of age and receipt antihypertensives to systolic blood pressure and shock index at injury scene and in the emergency department to predict massive transfusion in trauma patients

**DOI:** 10.1186/s13049-021-00840-2

**Published:** 2021-01-30

**Authors:** Se Jin Park, Mi Jin Lee, Changho Kim, Haewon Jung, Seong Hun Kim, Wooyoung Nho, Kang Suk Seo, Jungbae Park, Hyun Wook Ryoo, Jae Yun Ahn, Sungbae Moon, Jae Wan Cho, Shin-ah Son

**Affiliations:** 1 Department of Emergency Medicine, School of Medicine, Kyungpook National University, Daegu, Republic of Korea; 2 Department of Emergency Medicine, Gumi CHA Medical Center, CHA University, Gumi, Republic of Korea; 3 Department of Thoracic and Cardiovascular Surgery, School of Medicine, Kyungpook National University, Daegu, Republic of Korea

**Keywords:** Emergency medical services, Geriatrics, Antihypertensives, Massive transfusion, Shock index, Systolic blood pressure

## Abstract

**Background:**

Systolic blood pressure (SBP) and shock index (SI) are accurate indicators of hemodynamic instability and the need for transfusion in trauma patients. We aimed to determine whether the utility and cutoff point for SBP and SI are affected by age and antihypertensives.

**Methods:**

This was a retrospective observational study of a level 1 trauma center between January 2017 and December 2018. We analyzed the utility and cutoff points of SBP and SI for predicting massive transfusion (MT) and 30-day mortality according to patients’ age and whether they were taking antihypertensives. A multivariable logistic regression analysis was conducted to estimate the association of age and antihypertensives on primary and secondary outcomes.

**Results:**

We analyzed 4681 trauma cases. There were 1949 patients aged 65 years or older (41.6%), and 1375 hypertensive patients (29.4%). MT was given to 137 patients (2.9%). The 30-day mortality rate was 6.3% (*n* = 294). In geriatric trauma patients taking antihypertensives, a prehospital SBP less than 110 mmHg was the cutoff value for predicting MT in multivariate logistic regression analyses; packed red blood cell transfusion volume decreased abruptly based on prehospital SBP of 110 mmHg. Emergency Department SI greater than 1.0 was the cutoff value for predicting MT in patients who were older than 65 years and were not taking antihypertensives.

**Conclusions:**

The triage of trauma patients is based on the identification of clinical features readily identifiable by first responders. However, age and medications may also affect the accurate evaluation. In initial trauma management, we must apply SBP and SI differently depending on age, whether a patient is taking antihypertensives, and the time at which the indicators are measured.

**Supplementary Information:**

The online version contains supplementary material available at 10.1186/s13049-021-00840-2.

## Background

Trauma is an important cause of death among geriatric patients [1]. Older age is associated with higher rates of morbidity and mortality after injury as aging and the presence of comorbidities reduce physiological reserves [2, 3]. Age itself is a significant risk factor and predictor of increased mortality in polytrauma patients. A very low Glasgow Coma Scale (GCS) score and systolic blood pressure (SBP) of less than 80 mmHg are potential clinical indicators of massive bleeding and traumatic brain injury [4]. However, the assessment of injury severity and hemodynamic instability in geriatric patients is often difficult because of their altered response to injury [1, 5]. The low physiological capacity in geriatric patients masks the clinical exacerbation of injury, which makes the management of these patients more difficult [6].

SBP and heart rate (HR) are routinely used for assessing trauma patients [7, 8]. The shock index (SI), which is the ratio of HR to SBP, can be easily calculated in the field or emergency department (ED) [9]. SI has been an accurate indicator of hemodynamic instability and the need for transfusion in trauma patients [10] and serves as a marker for significant injury in trauma patients with hypovolemic shock, who likely needs massive transfusion (MT) [11]. However, it has been questioned whether SBP and HR are reliable for assessing geriatric trauma patients [6]. Early recognition of the need for MT is important but still presents a challenge in trauma patients [12].

Although SBP and SI are practical and useful predictors of outcomes for trauma patients, the literature has shown that SBP and SI cutoff points vary depending on the cause of trauma and the patient’s illness [9, 10, 13–16]. Under-triage among older injured patients is more likely to occur, with different thresholds for SBP, which indicates traumatic shock (< 90, < 100 or < 110 mmHg) [17]. Old age, hypertension, and β- or calcium channel blockers weaken the association between SI and 30-day mortality [13]. The role of SBP and SI as assessment tools in geriatric trauma patients or patients with antihypertensives is not well defined [18].

This study aimed to assess the utility and cutoff of points for SBP and SI assessed at the injury scene and in the ED for predicting MT and 30-day mortality in geriatric trauma patients and trauma patients taking antihypertensives.

## Methods

### Study design and population

**Fig. 1:**
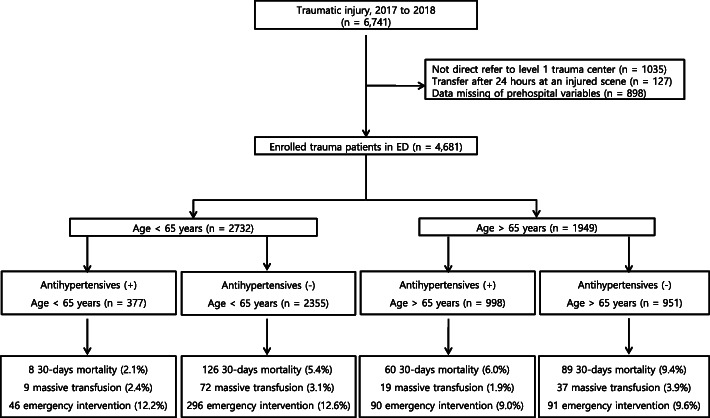
Study flow diagram of trauma stratified by age and antihypertensive use

This study was a retrospective, observational cross-sectional study. The data were obtained from the trauma database at Kyungpook National University Hospital, a regional level 1 trauma center in South Korea, from 1 January 2017 to 31 December 2018. We included patients who were admitted to the hospital after trauma who were 15 years old or older, and had a measurement of SBP and HR taken at the injury scene and in the ED. The exclusion criteria included patients who were transferred from other hospitals or using private vehicles, patients who had missing SBP or HR data, patients younger than 15 years old, patients who had incomplete registered data, and those who had burn injuries or trauma due to hanging (Fig. 1) [12, 14].

### Variables and data processing

We retrieved the detailed patient information including age, sex, vital signs, and comorbidities, such as diabetes mellitus, hypertension, congestive heart failure, cerebral vascular accident, and chronic renal disease. We documented trauma scores (e.g., Abbreviated Injury Scale, Revised Trauma Score [RTS], and Injury Severity Score [ISS]), units of packed red blood cells (PRBC) transfused within four and 24 h, and 30-day mortality. Elderly patients were defined as those who were 65 years old or older [19]. The prehospital and ED SIs were calculated as the ratio of HR to SBP in the field and ED as recorded by the emergency medical services personnel. ISS was classified into the following groups: 1 to 15, 16 to 24, and greater than 25 [20]. MT was defined as a requirement of 10 PRBCs or more in the first 24 h or 4 PRBCs or more in the first 4 h of hospital admission [21].

### Outcome measures

The primary outcome was MT and in-hospital mortality within 30 days. The secondary outcome was critical cutoff points of SBP (< 90, 90–110, and ≥ 110 mmHg) and SI as a categorical variable (< 0.7, 0.7–1, and ≥ 1) at the injury scene and in the ED [13, 14].

### Statistical analysis

The data were analyzed using IBM SPSS Statistics (version 25; IBM Corp., Armonk, NY, USA) and MedCalc (version 17; MedCalc Software, Mariakerke, Belgium). Categorical variables are presented as frequency and percent, whereas continuous variables are presented as mean ± standard deviation or as median and interquartile range (IQR, 25th–75th percentile). The Χ^2^ or Fisher’s exact tests were used to compare the categorical variables. The normality of the variables was determined using the Shapiro–Wilk test. Normally distributed data were expressed as mean and standard deviation, and median and IQRs were used for non-normally distributed data. A *t*- test or 1-way analysis of variance and the Mann–Whitney *U*- test were used to compare continuous variables.

Multivariable logistic regression analysis was used to identify the independent associations of univariate predictive variables on the occurrence of MT for adult trauma patients. Multivariable logistic regression analysis was performed in the age>65 years (geriatric group) and <65 years (young adult group), respectively. The odds ratio (OR) with 95% confidence interval (CI) of the associated conditions of the patients and the OR of a need for MT were based on vital signs at the injury scene and in the ED. A multivariable logistic regression analysis was conducted to estimate the association of age and antihypertensives on primary and secondary outcomes. Adjusted ORs were calculated after adjustment for sex, age, SBP and SI at the injury scene or at the initial time of ED admission, RTS, and ISS. *P*- values less than 0.05 were considered statistically significant.

## Results

### Overall characteristics of study patients

Of the 6741 trauma cases, 4681 patients were enrolled in this study. There were 1949 patients aged 65 years old or older (41.6%), and 1375 patients with hypertension (29.4%) (Fig. 1 and Online Supplemental Content). Two hundred twenty-two patients (4.7%) had received prehospital intravenous hydration. At the injury scene, the median SBP, HR, and SI were 130 mmHg, 84 beats per minute, and 0.7, respectively. On arrival at the ED, the median SBP, HR, and SI were 139 mmHg, 85 beats per minute, and 0.6, respectively. There were 914 (19.5%) transfused patients, and 137 (2.9%) had received MT. Other overall demographics are described in the Online Supplemental Content.

### Geriatric trauma patients according to antihypertensives and MT

**Fig. 2:**
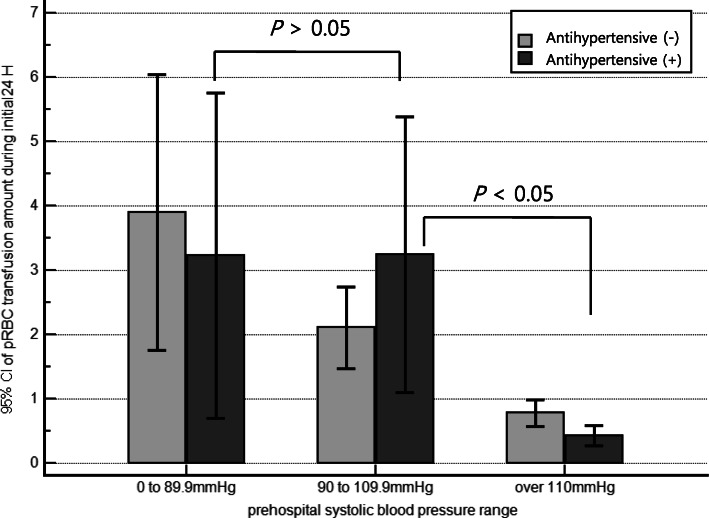
Red blood cell transfusion amount during the initial 24 h in subgroups of antihypertensives according to prehospital systolic blood pressure. *P values calculated by Kruskal–Wallis test with post-hoc analysis. The capped bars indicate 95% confidence intervals

Table 1 shows the general demographic information of the geriatric trauma population. Among 1949 elderly trauma patients, there were significant differences between antihypertensive and non-antihypertensive group in terms of age, sex, diabetes, SBP at the injury scene and in the ED, and SI on arrival in the ED. The median prehospital response time was the same in both groups (9 min) (Table 1). In the non-antihypertensive group, the total red blood cell transfusion volume gradually changed according to the prehospital SBP ranges. However, in the antihypertensive group, there was no difference between the SBP under 90 mmHg and SBP between 90 and 110 mmHg, and it decreased abruptly based on prehospital SBP 110 mmHg (Fig. 2).

**Table 1:** Baseline demographic characteristics of elderly trauma patients

	Overall elderly (***n*** = 1949)	Non-antihypertensive group (***n*** = 951)	Antihypertensive group (***n*** = 998)	***P*** value
Age (IQR), yr	76.0 (70.0–81.0)	75.0 (69.0–80.0)	77.0 (71.0–82.0)	< 0.001
Male sex, n (%)	1002 (51.4)	539 (56.7)	463 (46.4)	< 0.001
Comorbidities, n (%)				
Diabetes	529 (27.1)	167 (17.6)	362 (36.3)	< 0.001
Cerebrovascular accident	271 (13.9)	92 (9.7)	179 (17.9)	< 0.001
Congestive heart failure	68 (3.5)	29 (3.0)	39 (3.9)	0.575
Chronic renal disease	127 (6.5)	34 (3.6)	93 (9.3)	< 0.001
Traumatic brain injury, n (%)	96 (4.9)	57 (6.0)	39 (3.9)	0.033
Prehospital response time (IQR), min	9.0 (6.0–14.0)	9.0 (7.0–14.0)	9.0 (6.0–15.0)	0.912
Total prehospital time (IQR), min	24.0 (17.5–33.0)	25.0 (17.5–34.0)	23.0 (17.5–32.0)	0.498
Prehospital IV hydration, n (%)	57 (2.9)	38 (4.0)	19 (1.9)	0.014
Physiological parameters (IQR)				
At the injury scene				
SBP, mmHg	140.0 (121.0–160.0)	130.0 (120.0–153.0)	140.0 (120.0–160.0)	0.048
HR, beats per min	80.0 (72.0–90.0)	80.0 (72.0–90.0)	82.0 (72.0–93.5)	0.414
SI	0.59 (0.51–0.71)	0.60 (0.51–0.70)	0.59 (0.50–0.71)	0.571
At ED arrival				
SBP, mmHg	147.0 (124.0–165.0)	134.0 (121.0–162.0)	149.0 (128.0–168.0)	< 0.001
HR, beats per min	82.0 (71.0–94.0)	82.0 (71.0–95.0)	82.0 (71.0–94.0)	0.603
SI	0.57 (0.47–0.69)	0.58 (0.49–0.72)	0.56 (0.47–0.68)	0.001
GCS	15.0 (14.0–15.0)	15.0 (15.0–15.0)	15.0 (14.0–15.0)	0.131
Mechanism of injury, n (%)				0.433
Blunt	1916 (98.3)	933 (98.1)	983 (98.5)	
Penetrating	33 (1.7)	18 (1.9)	15 (1.5)	
ISS (IQR)	9.0 (5.0–14.0)	9.0 (5.0–16.0)	9.0 (5.0–13.0)	0.195
RTS (IQR)	12.0 (11.0–12.0)	12.0 (12.0–12.0)	12.0 (11.0–12.0)	0.004
Clinical course and interventions				
Length of stay in ICU (IQR), day	0 (0–0.67)	0 (0–0.65)	0 (0–0.67)	0.932
Transfusion, n (%)	443 (22.7)	210 (22.1)	233 (23.3)	0.794
Massive transfusion, n (%)	56 (2.9)	37 (3.9)	19 (1.9)	0.009
30-day mortality, n (%)	149 (7.6)	89 (9.4)	60 (6.0)	0.005

IQR denotes interquartile range

*ED* emergency department, *ICU* intensive care unit, *IV* intravenous, *HR* heart rate, *GCS* glasgow coma scale, *ISS* injury severity score, *RTS* revised trauma score, *SBP* systolic blood pressure, *SI* shock index

In geriatric trauma patients, there was no significant difference between the MT and non-MT groups in terms of age. The shock-related indices, GCS, and ISS were worse in the MT group (Table 2).

**Table 2:** Shock-related index between patients with and without massive transfusion in elderly trauma patients

	Overall elderly (***n*** = 1949)	Non–MT (***n*** = 1893)	MT (***n*** = 56)	***P*** value
Age (IQR), yr	76.0 (70.0–81.0)	76.0 (70.0–81.0)	76.0 (70.0–82.5)	0.766
Male sex, n (%)	1002 (51.4)	964 (50.9)	38 (67.9)	0.012
Taking antihypertensives, n(%)	998 (51.2)	979 (51.7)	19 (33.9)	0.009
Shock-related Index categories				
At injury scene (prehospital)				
SBP (IQR), mmHg	140.0 (121.0–160.0)	140.0 (120.0–160.0)	100.0 (100.0–120.0)	< 0.001
SI (IQR)	0.59 (0.51–0.71)	0.59 (0.51–0.69)	0.83 (0.65–0.90)	0.001
SBP category, n (%)				< 0.001
> 110 mmHg	1768 (90.6)	1742 (92.0)	27 (47.4)	
90–110 mmHg	149 (7.7)	125 (6.6)	24 (42.1)	
< 90 mmHg	33 (1.6)	27 (1.4)	6 (10.5)	
SI category, n (%)				< 0.001
< 0.7	1443 (73.9)	1422 (75.1)	21 (36.8)	
0.7–1.0	420 (21.7)	394 (20.8)	27 (47.4)	
> 1.0	86 (4.4)	78 (4.1)	9 (15.8)	
At ED arrival				
SBP (IQR), mmHg	147.0 (124.0–165.0)	151.0 (129.0–171.0)	88.0 (80.0–103.0)	< 0.001
SI (IQR)	0.57 (0.47–0.69)	0.55 (0.46–0.67)	0.94 (0.87–1.06)	< 0.001
SBP category, n (%)				< 0.001
> 110 mmHg	1692 (86.8)	1670 (88.2)	22 (38.9)	
90–110 mmHg	172 (8.8)	168 (8.9)	4 (7.4)	
< 90 mmHg	85 (4.3)	55 (2.9)	30 (53.7)	
SI category, n (%)				< 0.001
< 0.7	1468 (75.4)	1459 (77.1)	9 (16.7)	
0.7–1.0	372 (19.1)	354 (18.7)	18 (31.5)	
> 1.0	109 (5.5)	80 (4.2)	29 (51.9)	
GCS, n (%)				< 0.001
> 12	1778 (91.3)	1750 (92.3)	28 (50.0)	
9–12	68 (3.5)	62 (3.3)	6 (11.4)	
< 9	103 (5.1)	81 (4.3)	22 (38.6)	< 0.001
Injury severity score, n (%)				< 0.001
< 16	1482 (76.0)	1473 (77.8)	9 (16.1)	
16–24	292 (15.0)	267 (14.1)	25 (44.6)	
> 24	175 (9.0)	153 (8.1)	22 (39.3)	
30-day mortality, n (%)	149 (7.6)	121 (6.4)	28 (50.0)	< 0.001

IQR denotes interquartile range

*ED* emergency department, *GCS* glasgow coma scale, *SBP* systolic blood pressure, *SI* shock index

### Predicting clinical outcomes according to antihypertensives

Multivariable logistic regression analysis was conducted to compare the association between the shock-related index at the injury scene and in the ED and MT and 30-day mortality. In the univariate analysis for predicting MT, prehospital SBP of less than 110 mmHg, ED SI greater than 1.0, RTS, and ISS were the significant variables for elderly patients taking antihypertensives. However, prehospital SBP was the only strongly related factor among the aforementioned factors after multivariable analysis. In the elderly non-antihypertensive group, ED SI greater than 1.0 and RTS were statistically significant for predicting MT, but not prehospital SBP (Table 3A). As shown in Table 3B, ED SI greater than 1.0 significantly increased 30-day mortality only in the elderly non-antihypertensive group. But ISS significantly increased 30-day mortality in both groups.

**Table 3:** Multivariable logistic regression analysis for massive transfusion and 30-day mortality at the injury scene and emergency department admission in elderly trauma patients

	Aged > 65 years taking antihypertensives		Aged > 65 years not taking antihypertensives	
	Crude OR (95% CI)	Adjusted OR (95% CI)	Crude OR (95% CI)	Adjusted OR (95% CI)
A. Prediction of MT				
Male sex	**1.60** **(0.64–4.02)**	**2.51** **(0.09–72.7)**	**2.12** **(1.00–4.43)**	**0.68** **(0.16–2.99)**
Age, yr	**0.95** **(0.88–1.02)**	**1.13** **(0.91–1.40)**	**1.02** **(0.96–1.07)**	**0.99** **(0.88–1.11)**
Prehospital SBP				
< 90 mmHg	95.7^*^ **(4.78–194)**	94.0^*^ **(4.16–795)**	**6.38** **(0.67–61.1)**	**1.02** **(0.95–2.25)**
90–110 mmHg	28.7^*^ **(2.49–133)**	28.1^*^ **(1.75–52.0)**	10.8^*^ **(3.17–32.0)**	**2.37** **(0.48–11.7)**
> 110 mmHg	**1.00**	**1.00**	**1.00**	**1.00**
SI > 1.0. ED	20.7^*^ **(7.48–57.2)**	**8.04** **(0.17–83.8)**	23.9^*^ **(11.6–49.1)**	10.3^*^ **(2.27–46.3)**
RTS	0.52^*^ **(0.37–0.77)**	**0.35** **(0.10–1.16)**	0.33^*^ **(0.26–0.43)**	0.49^*^ **(0.28–0.87)**
ISS	1.09^*^ **(1.05–1.14)**	**1.08** **(0.96–1.22)**	1.10^*^ **(1.06–1.14)**	**1.02** **(0.93–1.12)**
B. 30-day mortality				
Male sex	**1.01** **(0.60–1.71)**	**0.29** **(0.07–1.25)**	2.09^*^ **(1.29–3.37)**	**1.00** **(0.31–3.28)**
Age, yr	**1.00** **(0.96–1.03)**	**1.08** **(0.98–1.19)**	**1.00** **(0.97–1.03)**	**0.99** **(0.92–1.08)**
Prehospital SBP				
< 90 mmHg	**5.00** **(0.50–50.5)**	**1.00** **(0.06–12.0)**	**4.56** **(0.80–26.1)**	**1.01** **(0.26–11.0)**
90–110 mmHg	**1.50** **(0.33–6.93)**	**2.69** **(0.44–16.6)**	2.88^*^ **(1.06–7.85)**	**0.79** **(0.17–3.72)**
> 110 mmHg	**1.00**	**1.00**	**1.00**	**1.00**
SI > 1.0. ED	7.96^*^ **(3.61–17.5)**	**0.80** **(0.04–17.1)**	3.05^*^ **(1.51–6.17)**	0.06^*^ **(0.01–0.56)**
RTS	0.30^*^ **(0.22–0.39)**	0.45^*^ **(0.28–0.71)**	0.28^*^ **(0.22–0.36)**	0.33^*^ **(0.21–0.53)**
ISS	1.18^*^ **(1.14–1.22)**	1.12^*^ **(1.05–1.20)**	1.15^*^ **(1.21–1.18)**	1.09^*^ **(1.02–1.16)**

*ED* emergency department, *CI* confidence interval, *ISS* Injury Severity Score, *MT* massive transfusion, *OR* odds ratio, *RTS* Revised Trauma Score, *SBP* systolic blood pressure, *SI* shock index

*P value < 0.05

### Predicting MT stratified by age and antihypertensives

**Fig. 3:**
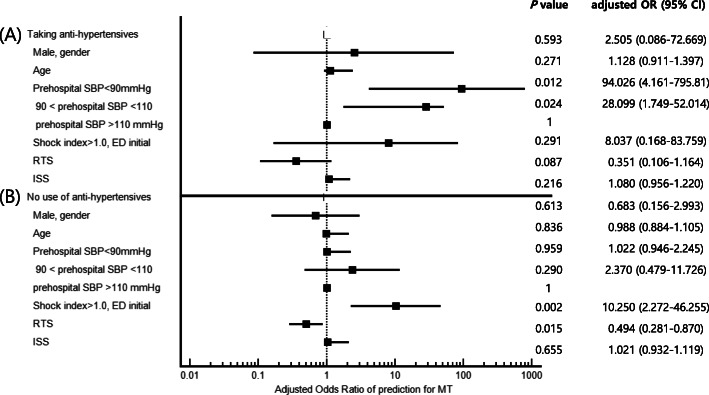
Forest plot multivariable logistic regression analysis for massive transfusion prediction in trauma patients at the injury scene and at the time of emergency department admission in elderly trauma patients

We conducted multivariable logistic regression analyses of the associated factors that were significant in the univariate logistic regression analyses for MT prediction (Table 4). In the subgroup analysis for MT stratified by age and antihypertensives, only prehospital SBP was a significant predicting factor in elderly trauma patients taking antihypertensives (Fig. 3A). However, ED SI greater than 1.0 and RTS were statistically significant in the elderly patients not taking antihypertensives (Fig. 3B).

**Table 4:** Multivariable logistic regression for predicting massive transfusion stratified by age and antihypertensives

	Aged > 65 years not taking antihypertensives		Aged > 65 years taking antihypertensives		Aged < 65 years not taking antihypertensives		Aged < 65 years taking antihypertensives	
	*P* value	aOR (95% CI)	*P* value	aOR (95% CI)	*P* value	aOR (95% CI)	*P* value	aOR (95% CI)
Male sex	0.613	**0.68** **(0.16–2.99)**	0.593	**2.51** **(0.09–72.6)**	0.997	**1.00** **(0.29–3.38)**	0.108	**0.14** **(0.01–1.55)**
Age, yr	0.836	**0.99** **(0.88–1.11)**	0.271	**1.13** **(0.91–1.40)**	0.039	1.04^*^ **(1.00–1.07)**	0.909	**0.99** **(0.86–1.15)**
Prehospital SBP								
< 90 mmHg	0.959	**1.02** **(0.95–2.25)**	0.012	94.0^*^ **(4.16–795)**	0.237	**2.12** **(0.61–7.23)**	0.441	**4.16** **(0.11–36.6)**
90–110 mmHg	0.290	**2.37** **(0.48–11.7)**	0.024	28.1^*^ **(1.75–52.0)**	0.447	**1.58** **(0.49–5.12)**	0.049	10.3^*^ **(1.01–44.7)**
> 110 mmHg (reference)	–	**1.00**	–	**1.00**	–	**1.00**	–	**1.00**
SI > 1.0. ED initial	0.002	10.3^*^ **(2.27–46.3)**	0.291	**8.04** **(0.17–83.8)**	0.044	3.87^*^ **(1.36–11.0)**	0.802	**0.67** **(0.03–15.1)**
RTS	0.015	0.49^*^ **(0.28–0.87)**	0.087	**0.35** **(0.11–1.16)**	0.001	0.59^*^ **(0.43–0.81)**	0.147	**0.52** **(0.22–1.26)**
ISS	0.655	**1.02** **(0.93–1.12)**	0.216	**1.08** **(0.96–1.22)**	< 0.001	1.10^*^ **(1.05–1.14)**	0.100	**1.06** **(0.99–1.13)**

Adjusted for sex, age, prehospital SBP, SI measured at ED admission, RTS, and ISS

*aOR* adjusted odds ratio, *CI* confidence interval, *ED* emergency department, *ISS* Injury Severity Score, *RTS* Revised Trauma Score, *SBP* systolic blood pressure, *SI* shock index

*P value < 0.05

## Discussion

We aimed to examine the utility and cutoff of points for SBP and SI, as determined at the injury scene and in the ED, for predicting MT and 30-day mortality in geriatric trauma patients and patients taking antihypertensives. In geriatric trauma patients taking antihypertensives, a prehospital SBP lower than 110 mmHg was the cutoff value for the prediction of MT in multivariable logistic regression analyses. There was no significant value in multivariable logistic regression analyses of prehospital SI for predicting MT and 30-day mortality. ED SI greater than 1.0 was the cutoff value for predicting MT in patients who were older than 65 years and who were not taking antihypertensives.

The European guideline on the management of major bleeding and coagulopathy after trauma recommends that physicians clinically assess the extent of traumatic hemorrhage using a combination of patient physiology, anatomical injury pattern, mechanism of injury, and patient response to initial resuscitation. Isolated vital signs, such as HR or SBP, are unreliable in the assessment of hypovolemic shock. HR alone does not predict the need for MT [11, 22]. In contrast, SI has been shown to better risk-stratify patients for critical bleeding, increased transfusion requirements and early mortality [11, 23]. SI is an effective tool that can be implemented in a prehospital setting for the triage of geriatric trauma patients and is superior to HR and SBP for predicting mortality in geriatric trauma patients. Geriatric trauma patients with an SI of 1 or greater should be transferred to a Level 1 trauma center [6]. Bruijns et al. reported that the difference between ED and prehospital SBP, respiratory rate, and SI are good predictor of 48-h mortality in trauma and may supplement decisions on trauma treatment [24]. Fligor et al. reported that MT in geriatric trauma patients can be reliably and simply predicted by the arrival vital signs of SBP, pulse pressure, diastolic blood pressure, and SI [22]. Most studies have assessed predictive properties of SI for requiring at least 10 units of PRBC in the first 24 h. It would be useful to conduct more studies evaluating the estimation and recording of the SI at earlier time-points, including the prehospital phase [25].

MT in older trauma patients should be considered based on anatomical factors, pre-injury anticoagulant or antiplatelet agent use, lactate level and SI even if traditional vital signs are normal [26]. Studies have suggested that advanced age and the presence of comorbidities such as hypertension, diabetes, use of anticoagulation medications can affect the prognosis of trauma patients based on SBP and SI, and therefore the cutoff value should be different. Kristensen et al. reported that SI is independently associated with 30-day mortality in a broad population of ED patients, and an SI of 1 or more is associated with an adjusted OR of 10.5 (95% CI, 9.3–11.7) for 30-day mortality. However, age, hypertension, and β- or calcium channel blockers weaken the association between SI and mortality [13]. Additionally, Brown et al. [27] noted that formally substituting an SBP of less than 110 mmHg for the current SBP of less than 90 mmHg is beneficial in geriatric patients and may be potentially valuable in all adult patients at field triage. Geriatric patients with SBP of 90 to 109 mmHg had odds of mortality similar to those of geriatric patients with SBP of less than 90 mmHg [27]. SBP of less than 110 might represent shock in patients older than 65 years [28].

Prehospital trauma triage ensures proper transport of patients at risk of severe injury to hospitals with an appropriate level of trauma care. Incorrect triage results in under-triage and over-triage [29]. According to the National Trauma Triage Protocol guideline [30], GCS, SBP and respiratory rate are involved in Step 1 of field triage. Next, the anatomy of injury is considered in Step 2; the mechanism of injury is considered in Step 3; and older age, anticoagulant use, and bleeding disorders are considered in Step 4. Studies of trauma patients have suggested an upward review of the SBP cutoff value to less than 90 mmHg in elderly patients older than 65 years [8, 16, 27]. The above are reflected in the National Trauma Triage Protocol guideline in Step 4, and the presence or absence of antihypertensive use is not considered. Based on this and other studies, it is advisable to include antihypertensive use as a consideration in field triage and to consider it at an earlier stage along with older age [27]. For example, we recommend that “SBP < 110 might represent shock after age 65 years” is eliminated on Step 4 and “SBP < 110 might represent shock after age 65 years taking antihypertensives” is added on Step 2.

SBP, HR and SI, the main analysis variables we used, are continuous variables, and some results may be different depending on the interval classification method. When they were used as a continuous variable instead of an interval for MT prediction, SI at ED arrival [odds ratio 257 (95% CI 8.36–791) in elderly patients taking antihypertensives, odds ratio 20.9 (95% CI 4.04–108) in elderly patients not taking antihypertensives] was analyzed as a statistically significant factor along with ISS and RTS. However, in this case, the influence of SI unit that is smaller than SBP unit, increase in the unit and interval characteristics of the continuous variable seen in regression analysis. So unit correction is required. And we assumed that presenting the cut-off value is familiar to clinician and more useful for interpretation.

We analyzed both the patients’ medical history and prehospital vital signs and indicators, which were not fully covered in previous studies, and performed multivariable logistic regression analysis including in-hospital mortality and MT. In addition, we were able to present a more accurate understanding of the underlying condition and flexible cutoff by stratifying the associations of vital signs and SI on clinical prognosis according to age and whether the patient was taking antihypertensives. In the group of patients who were younger than 65 years and not taking antihypertensives, RTS, ISS and ED SI were strongly associated with prediction of MT. Prehospital SBP was the only significant predicting factor in elderly trauma patients taking antihypertensives. However, SI measured at the time of ED admission and RTS were significant in the elderly patients who were not taking antihypertensives. These results could suggest that the uncritical application by cutoff values of SI and SBP in any aged trauma patients is inaccurate.

### Limitations

Our study had several limitations. Because this was an observational study, there was a potential selection bias from excluding many patients who were missing or did not have SBP and HR measured in the emergency medical services setting (*n* = 898, Fig. 1). These were 684 patients not transported by EMS or direct visits (including patients under the age of 14), 269 patients under the age of 14, and 26 cases of refusal to measure vital sign because of self-harm, etc. Instead, their ED vital signs are all recorded (95% CI of ED SBP 120–156 mmHg, ED HR 74–98 bpm). Second, these contradictory results included a potential bias in that no transfusion was recorded if a patient died before receiving a transfusion, despite being a severe trauma patient requiring transfusion. Therefore, although MT is the main interest, we also studied the 30-day-mortality as a primary outcome. Finally, we did not exclude traumatic brain injury, which may confound the data due to the classic Cushing reflex whereby SBP increases and HR decreases. However, the predictive effect of SI and SBP was not changed in the primary outcome for the remaining 1853 patients excluding 96 severe traumatic brain injury (TBI) patients.

## Conclusions

We need to apply the indicators differently depending on a patient’s age and the presence of hypertension in the initial trauma management. In trauma patients older than 65 years who are taking antihypertensives, MT can be predicted based on SBP of 110 mmHg measured in the field, whereas in patients older than 65 years and not using antihypertensives, MT can be predicted based on an ED SI of 1.0. Further research is needed to establish the vital signs and delta SI at the injury scene and ED discharge in elderly trauma patients.

## Supplementary Information

**Additional file 1:** **Supplement 1**. Demographics of enrolled trauma patients. **Supplement 2**. Baseline demographic characteristics of younger trauma patients.

## Data Availability

Not applicable.

## Abbreviations

CI: Confidence interval
ED: Emergency department
ISS: Injury Severity Score
KNUH: KNUH University Hospital
OR: Odds ratio
PRBC: Packed red blood cells
RTS: Revised Trauma Score
GCS: Glasgow Coma Scale
HR: Heart rate
MT: Massive transfusion
SBP: Systolic blood pressure
SI: Shock index
