# Supplementary material for: The impact of age and receipt antihypertensives to systolic blood pressure and shock index at injury scene and in the emergency department to predict massive transfusion in trauma patients
Source: Scand J Trauma Resusc Emerg Med. 2021 Jan 30;29:26. doi: 10.1186/s13049-021-00840-2 (PMC7847168; doi:10.1186/s13049-021-00840-2)
Supplement: Supplementary file 1 — Additional file 1: Supplement 1. Demographics of enrolled trauma patients. Supplement 2. Baseline demographic characteristics of younger trauma patients. [file 13049_2021_840_MOESM1_ESM.docx]

**Supplement 1.** Demographics of enrolled trauma patients

| **Variables** |  | **Total (n = 4681)** |
| --- | --- | --- |
| Age (IQR), yr |  | 60.0 (44.0–74.0) |
| Male sex, n (%) |  | 3061 (65.4) |
| Comorbidities, n (%) |  |  |
| Diabetes |  | 806 (17.2) |
| Hypertension |  | 1375 (29.4) |
| Cerebrovascular accident |  | 343 (7.3) |
| Congestive heart failure |  | 73 (1.6) |
| Chronic renal disease |  | 160 (3.4) |
| Traumatic brain injury, n (%) |  | 230 (4.9) |
| Prehospital IV hydration, n (%) |  | 222 (4.7) |
| Prehospital transport time (IQR), min |  | 25 (20–38) |
| Physiological parameters (IQR)  At the injury scene |  |  |
| SBP, mmHg |  | 130 (110–142.3) |
| HR, beats per min |  | 84 (76-96) |
| SI |  | 0.7 (0.6–0.8) |
| At ED arrival |  |  |
| SBP, mmHg |  | 139 (120–1580) |
| HR, beats per min |  | 85 (74–98) |
| SI |  | 0.6 (0.5–0.8) |
| Mechanism of injury, n (%) |  |  |
| Blunt |  | 4464 (95.4) |
| Penetrating |  | 190 (4.1) |
| ISS (IQR) |  | 9 (4–14) |
| RTS (IQR) |  | 12 (12–12) |
| Clinical course and related interventions |  |  |
| Length of stay in ICU (IQR), day |  | 0 (0–0.7) |
| Emergency operation or intervention, n (%) |  | 523 (11.2) |
| Transfusion, n (%) |  | 914 (19.5) |
| Massive transfusion, n (%) |  | 137 (2.9) |
| Hospital mortality, n (%) |  | 294 (6.3) |

IQR denotes interquartile range.

ED, emergency department; ICU, intensive care unit; HR, heart rate; ISS, Injury Severity Score; IV, intravenous; RTS, Revised Trauma Score; SBP, systolic blood pressure; SI, shock index

**Supplement 2.** Baseline demographic characteristics of younger trauma patients

|  | **Overall the young**  **(n = 2732)** | **Non-antihypertensive group**  **(n = 2355)** | **Antihypertensive group**  **(n = 377)** | ***P* value** |
| --- | --- | --- | --- | --- |
| Age (IQR), yr | 48.0 (31.0–57.0) | 45.0 (28.0–56.0) | 58.0 (51.0–61.0) | <0.001 |
| Male sex, n (%) | 2059 (75.4) | 1761 (74.8) | 298 (79.0) | <0.074 |
| Comorbidities, n (%) |  |  |  |  |
| Diabetes | 277 (10.1) | 153 (6.5) | 124 (32.9) | <0.001 |
| Cerebrovascular accident | 72 (2.6) | 46 (2.0) | 26 (6.9) | <0.001 |
| Congestive heart failure | 5 (0.2) | 2 (0.1) | 3 (0.8) | <0.001 |
| Chronic renal disease | 33 (1.2) | 13 (0.6) | 20 (5.3) | <0.001 |
| Traumatic brain injury, n (%) | 134 (4.9) | 121 (5.1) | 13 (3.4) | 0.158 |
| Prehospital response time (IQR), min | 9.0 (6.0–14.0) | 9.0 (7.0–14.0) | 9.0 (6.0–15.0) | 0.912 |
| Total prehospital time (IQR), min | 24.0 (17.5–33.0) | 25.0 (17.5–34.0) | 23.0 (17.5–32.0) | 0.498 |
| Prehospital IV hydration, n (%) | 165 (6.0) | 142 (6.0) | 23 (6.1) | 0.057 |
| Physiological parameters (IQR) |  |  |  |  |
| At the injury scene |  |  |  |  |
| SBP, mmHg | 120.0 (110.0–140.0) | 120.0 (110.0–132.0) | 130.0 (107.5–150.0) | 0.002 |
| HR, beats per min | 85.0 (78.0–98.0) | 85.0 (78.0–98.0) | 85.0 (72.0–96.0) | 0.150 |
| SI | 0.59 (0.51–0.71) | 0.7 (0.61–0.83) | 0.67 (0.55–0.78) | 0.571 |
| At ED arrival |  |  |  |  |
| SBP, mmHg | 135.0 (118.0–152.0) | 134.0 (117.0–151.0) | 140.0 (120.0–159.3) | <0.001 |
| HR, beats per min | 87.0 (76.0–100.0) | 88.0 (76.0–101.0) | 85.0 (74.0–96.0) | 0.002 |
| SI | 0.65 (0.55–0.78) | 0.66 (0.55–0.79) | 0.61 (0.51–0.73) | <0.001 |
| GCS | 15.0 (15.0–15.0) | 15.0 (15.0–15.0) | 15.0 (15.0–15.0) | 0.910 |
| Mechanism of injury, n (%) |  |  |  | 0.102 |
| Blunt | 2548 (93.3) | 2189 (93.0) | 359 (95.2) |  |
| Penetrating | 184 (6.7) | 166 (7.0) | 18 (4.8) |  |
| ISS (IQR) | 9.0 (4.0–14.0) | 9.0 (4.0–14.0) | 9.0 (4.0–16.0) | 0.004 |
| RTS (IQR) | 12.0 (12.0–12.0) | 12.0 (12.0–12.0) | 12.0 (12.0–12.0) | 0.407 |
| Clinical course and interventions |  |  |  |  |
| Length of stay in ICU (IQR), day | 0 (0–0.76) | 0 (0–0.69) | 0 (0–1.62) | 0.085 |
| Transfusion, n (%) | 471 (17.2) | 387 (16.4) | 84 (22.3) | 0.005 |
| Massive transfusion, n (%) | 81 (3.0) | 72 (3.1) | 9 (2.4) | 0.476 |
| 30-day mortality, n (%) | 134 (4.9) | 126 (5.4) | 8 (2.1) | 0.007 |

IQR denotes interquartile range.

ED, emergency department; ICU, intensive care unit; IV, intravenous; HR, heart rate; GCS, glasgow coma scale; ISS, injury severity score; RTS, revised trauma score; SBP, systolic blood pressure; SI, shock index
